# Vegetation water use efficiency constrains the dynamic of net primary productivity in Mu Us Sandy Land

**DOI:** 10.3389/fpls.2026.1724283

**Published:** 2026-02-04

**Authors:** Yunfei Chen, Zuyu Liu, Xinjia Guo, Kun Liu, Leyi Zhang, Jing Liu, Xiuhua Liu, Junqi He

**Affiliations:** 1 Key Laboratory of Subsurface Hydrology and Ecological Effect in Arid Region of Ministry of Education, School of Water and Environment, Chang’an University, Xi’an, China; 2 Key Laboratory of Eco-hydrology and Water Security in Arid and Semi-arid Regions of Ministry of Water Resources, Chang’an University, Xi’an, China; 3 College of Hydraulic and Civil Engineering, Xinjiang Agricultural University, Urumqi, China

**Keywords:** vegetation restoration, climate change, environmental factors, precipitation use efficiency, arid ecosystems

## Abstract

The Mu Us Sandy Land (MUSL) in Northwest China, once one of the world’s most severely desertification areas, is now a leading example of successful ecological restoration. Undoubtedly, ecological restoration efforts have significantly reversed this region’s desertification and enhanced carbon sequestration. However, uncertainties remain regarding whether ongoing and expanded restoration initiatives can continue to stabilize or further enhance the productivity and carbon sequestration capacity of the region’s ecosystems. Net primary productivity (NPP) as an important indicator for evaluating ecosystem function and sustainability. Analyzing its spatial-temporal evolves differences and driving mechanisms is crucial for formulating effective ecological restoration policies. Given this, this study applied the Breaks for Additive Season and Trend (BFAST) method to diagnose the NPP’s spatiotemporal dynamics and development patterns in the MUSL from 2001 to 2020. The results revealed significant temporal-spatial variations in NPP, identifying eight development patterns (Type1-8). Among these, three increasing trends patterns—Type1, Type3, and Type5—were dominant, driven by rising regional precipitation and temperature, accounting for 82.9% of the total areas. In contrast, in the southern area with more favorable hydrothermal conditions, Type7 (from increase to decrease) was dominated, accounting for 16.4%. NPP in this region initially increased from 52 gC·m^-2^ to 117 gC·m^-2^, before declining to 70 gC·m^-2^, reflecting a notable decline. The structural equation model’s results indicated that although the high synergistic effect between climatic factors and ecological restoration significantly promoted the rapid increase of NPP in most areas of the MUSL, it might not alter the declining NPP in the southern regions (Type7) with more favorable hydrothermal conditions, where vegetation shows low response to precipitation. While further analysis indicated that reaching the threshold of water use efficiency is one of a significant reason for the decline in NPP in the southern region. Overall, these findings and insights not only provide reliable data and guidance for future ecological restoration projects, but also serve as a crucial warning for vegetation management in arid regions.

## 1 Introduction

Dryland ecosystems, which cover approximately 40% of the Earth’s terrestrial area, are an essential component of the global ecosystem (Schlaepfer et al., 2017; Maestre et al., 2024). They not only play a critical role in maintaining biodiversity, regulating the climate change and contributing to carbon neutrality, while also providing a wide range of essential ecosystem services that support human survival (Gross et al., 2017; Yao et al., 2020; Hu et al., 2021). However, due to the inherently fragile environmental conditions and their high sensitivity to climate change in arid region, the structural stability of dryland ecosystems and the long-term effectiveness of ecological restoration efforts remain seriously threatened (Ma et al., 2022). These challenges have driven growing research interest in ecosystem resilience and the long-term sustainability of management interventions (Dem et al., 2025). Therefore, establishing scientifically robust evaluation frameworks capable of accurately capturing ecosystem dynamics has become a critical focus in ecological governance research. Net primary productivity (NPP), a key indicator of vegetation carbon sequestration and productivity levels, is essential in evaluating the effectiveness of ecosystem restoration (Wang et al., 2020; Ye et al., 2020). Tracking the spatiotemporal evolution of NPP enables a deeper understanding of ecological response mechanisms and provides a scientific basis for evaluating long-term ecosystem sustainability.

In this context, increasing attention has been directed towards understanding the complex mechanisms through which multiple drivers jointly shape NPP dynamics over time (Hunt et al., 2024; Terry and Adler, 2024). To date, numerous studies have demonstrated that climatic variables, by altering environmental conditions, have a profound impact on plant structural and functional processes, thus serving as the primary drivers of NPP variability (Grunzweig et al., 2022; Qiu et al., 2024). Among these, temperature and precipitation patterns are key determinants of NPP—favorable thermal and moisture conditions enhance vegetation productivity, whereas extreme drought and heat events can significantly reduce NPP (Jiang et al., 2018). As for human activities, their impact on NPP enhancement remains a subject of ongoing debate (Wei et al., 2022). Numerous studies have demonstrated that large-scale, high-density, and intensive ecological restoration projects, such as afforestation, grassland restoration, and the conversion of farmland to forest, can significantly increase NPP in the short term by altering ecosystem structure, processes, and functions (Liu et al., 2019; Lv et al., 2024). In contrast, activities such as overgrazing, urban expansion, and mineral resource development may have negatively influence on land use change and local terrestrial ecosystems, leading to a decline in NPP (Xu et al., 2025). However, although NPP is influenced by multiple mechanisms involving both climatic factors and human activities, most studies have primarily concentrated on quantifying and distinguishing the individual effects of these factors, neglecting their potential synergies (Feng et al., 2021). As a result, the coupling effects of climate factors and human activities on NPP dynamics in ecosystems remain insufficiently understood. In particular, the quantitative analysis of NPP evolution characteristics and their driving mechanisms under multi-development pattern requires further investigation.

Despite the widespread use of traditional statistical methods (e.g., correlation analysis and regression models), trend decomposition techniques (e.g., empirical mode decomposition), Mann-Kendall (MK) mutation detection, residual analysis, and geographical detectors to investigate NPP variation patterns and influencing factors (Wang et al., 2020; Gao et al., 2022), these approaches still have limitations in revealing multi-factor synergies, nonlinear relationships, and structural changes under strong seasonal backgrounds (Zhang et al., 2023). For instance, single linear regression may obscure vegetation browning by averaging it with overall greening trends (Jiang et al., 2020a). The MK method primarily focuses on monotonic trends or discrete mutation points, making it challenging to separate seasonal components from long-term trends and accurately identify breakpoints magnitudes (Zhang and Wu, 2020). Meanwhile, residual trend analysis assesses the impact of human activities on vegetation by calculating the residuals from multivariate regression analysis of climatic factors and vegetation indices, yet it fails to capture complex nonlinear interactions between variables (Rhif et al., 2022). In contrast, the Breaks for Additive Season and Trend (BFAST) algorithm, proposed by Verbesselt et al. (2010) offers greater adaptability, enabling the detection of multiple change types—seasonal, gradual, and abrupt—in remote sensing time series (Dou et al., 2024). As an improved version of BFAST, BFAST01’s core functionality lies in its ability to precisely identify the most significant breakpoints within a trend, allowing the overall trend to be divided into two distinctly different phases rather than multiple smaller segments (Wang et al., 2023a, 2024). This feature enables BFAST01, compared to BFAST, to detect local breakpoints hidden beneath the overarching trend at the regional scale, thus providing new possibilities for multi-factor, multi-scale ecological dynamics analysis (Mardian et al., 2021; Rhif et al., 2022). Although BFAST01 has shown immense potential for applications in remote sensing and ecology, its use remains limited, particularly in the study of NPP’s nonlinear dynamics in arid regions, where its application has not been fully explored.

Given the long-term ecological implications of vegetation restoration in dryland regions, it is urgent to evaluate the development patterns, dynamic trajectories, and driving factors of NPP under restoration. This evaluation will help identify stability trends and provide a scientific basis for informed ecological management and timely interventions in the face of potential risks. The Mu Us Sandy Land (MUSL) in Northwest China is an ideal case study, as it was once one of the most severely desertification areas in the world but now it has become one of the most successful regions for ecological restoration (Chen et al., 2022; Zhou et al., 2023). The implementation of environmental protection programs has undoubtedly improved the MUSL’s ecological environment, increased species diversity, and significantly enhanced ecosystem productivity and regional carbon sequestration (Zhou et al., 2021). However, large-scale vegetation reconstruction may introduce new trade-offs and challenges to the region’s water resources and carbon balance (Zhao et al., 2020; Wang et al., 2023a). A clear example of this is observed in the southern part of the MUSL, where the Normalized Difference Vegetation Index (NDVI) has shifted from a previous upward trend to a consistent decline since 2011 (Gao et al., 2022). This shift may further indicate vegetation saturation or emerging resource limitations associated with intensified restoration efforts. Nevertheless, it remains uncertain whether current and further expanded ecological restoration efforts can continue to stabilize or enhance the productivity and carbon sequestration capacity of dryland ecosystems, and whether potential issues associated with post-restoration high-density vegetation have been overlooked. These questions remain insufficiently addressed.

The primary objective of this study, therefore, is to evaluate the effectiveness of ecological restoration programs by focusing on the dynamics of vegetation productivity since the implementation of multiple projects (2000–2020). To achieve this, a comprehensive methodological framework was employed, incorporating the BFAST01 algorithm, Structural Equation Modeling (SEM), and ecosystem efficiency indicators such as water use efficiency (WUE=NPP/ET) and precipitation use efficiency (PUE=NPP/P). The specific objectives are: (1) to identify diverse NPP development patterns (including increases, declines, and abrupt changes) during ecological restoration, and to quantitatively assess the contribution of each development type to vegetation recovery; (2) to determine the dominant drivers of each NPP pattern and evaluate the direct, indirect, and synergistic effects of climate change and human-induced restoration activities; and (3) to evaluate the effectiveness of current ecological management strategies and delineate priority areas for optimizing ecosystem management and artificial interventions. By identifying potential ecological risks, this study provides continuous, data-driven support for desertification control, sustainability assessment, and mechanism analysis, thereby offering a theoretical basis for ecological restoration and afforestation programs on the Loess Plateau and in other ecologically fragile dryland regions.

## 2 Materials and methods

### 2.1 Study area

**Figure 1:**
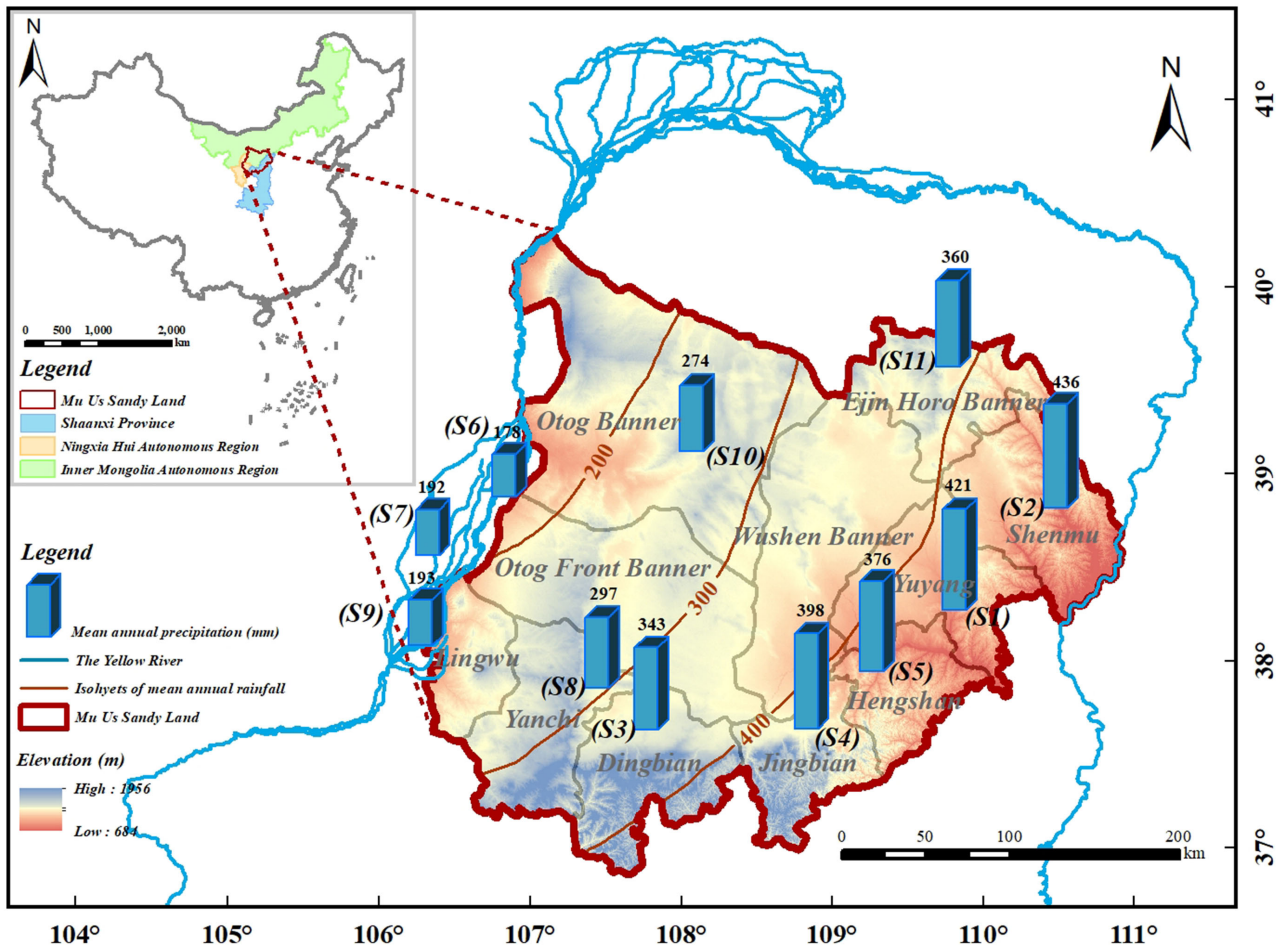
Geographical location of the Mu Us Sandy Land and spatial distribution of 11 meteorological stations (S1–S11).

The MUSL, one of the four major sandy lands in China—alongside the Horqin, Hulunbuir, and Hunsandake Sandy Land—covers an area of approximately 91,200 km^2^. It is bounded by the Yellow River on three sides and lies at the junction of Shaanxi Province, Inner Mongolia Autonomous Region, and Ningxia Hui Autonomous Region (36°49′–40°N, 106°11′–110°54′E). The terrain is relatively flat, with elevations ranging from 684 to 1,956 meters, while slightly higher altitudes (1,600–1,700 meters) are found in the southern part (Ma et al., 2019). The study area encompasses 11 national meteorological stations distributed across the following counties and districts (**Figure 1**): (1) Shaanxi Province: Yuyang District (S1), Shenmu County (S2), Dingbian County (S3), Jingbian County (S4), and Hengshan County (S5); (2) Ningxia Hui Autonomous Region: Taole county (S6), Yinchuan (S7), Yanchi County (S8), and Wuzhong (S9); (3) Inner Mongolia Autonomous Region: Otog Banner (S10) and Ejin Horo Banner (S11).

In terms of hydroclimatic conditions, the MUSL experiences a typical temperate continental climate and is situated at the boundary between semi-arid and semi-humid regions. Based on meteorological records from 11 national stations between 1982 and 2020 (**Figure 1**), the annual precipitation ranges from 170 to 440 mm, with a clear southeast–northwest decreasing gradient. Rainfall is markedly seasonal, with 60% to 75% occurring from July to September. Sunlight is abundant, with annual sunshine hours decreasing from 3,000–3,100 hours in the northwest to 2,700–2,900 hours in the southeast. Correspondingly, total annual solar radiation declines from approximately 6,270 MJ/m^2^ to 5,770 MJ/m^2^. The annual mean temperature ranges from 7.2 to 10.4°C, also gradually decreasing from southeast to northwest. Intra-annual temperature variation is pronounced, with January averages ranging from −10 to −6°C and July averages from 22 to 24°C (Chen et al., 2024a). In addition to climatic gradients, the MUSL exhibits distinct ecological transitions in vegetation and soil. From northwest to southeast, the region can be divided into three major zones: the brown calcic soil semi-desert zone, the gray calcic soil desert steppe zone, and the gray cinnamon soil forest-steppe zone (Feng et al., 2021).

### 2.2 Datasets and pre-processing

#### 2.2.1 NPP data set

The NPP data used in this study were obtained from the MOD17A3 dataset, provided by NASA’s EOS/MODIS platform, covering the period from 2001 to 2020. These data are publicly available and can be downloaded from the official website (https://lpdaac.usgs.gov/; **Table 1**). The MOD17A3 product estimates annual terrestrial vegetation NPP globally, based on MODIS sensor observations and calculations derived from the Biome-BGC model. To enhance data reliability, the dataset includes corrections for cloud contamination and aerosol interference, along with annual quality control information (NPP_QC). A comprehensive analysis of the NPP_QC layer for the MUSL was conducted in this study, categorizing the data quality into four levels: high, medium, low, and retrieval failure. The results showed that over the observation period, the combined proportion of high- and medium-quality pixels exceeded 85%, indicating the overall reliability and suitability of the dataset for ecological studies in the MUSL. Here, the MOD17A3 NPP products from 2001 to 2020, with a native spatial resolution of 500 meters, were preprocessed for this study. The data were mosaicked, reprojected, and resampled using the MODIS Reprojection Tool (MRT) to maintain a consistent 500-meter resolution. Subsequently, annual NPP raster layers were clipped to the study area boundary using ArcGIS to extract NPP values for the MUSL from 2001 to 2020.

**Table 1:** The Detailed information of data sources.

Data set	Time	Resolution ratio	Source from
NPP	2001~2020	500 m	https://ladsweb.modaps.eosdis.nasa.gov/
ET	2001~2020	500 m	https://ladsweb.modaps.eosdis.nasa.gov/
T&P	2001~2020	—	https://data.cma.cn/
SM	2001~2020	1000 m	https://data.tpdc.ac.cn/
VPD	2001~2020	4400 m	https://climate.northwestknowledge.net/
SR	2001~2020	10 km	https://cds.climate.copernicus.eu/
DEM	—	30 m	https://www.gscloud.cn/
Slope	—	30 m	https://www.gscloud.cn/
Aspect	—	30 m	https://www.gscloud.cn/
SPEI	2001~2020	1000 m	https://doi.org/10.57760/sciencedb.ecodb.00090
LULC	2000, 2010, 2020	30 m	https://www.resdc.cn/

NPP, Net Primary Product; ET, Evapotranspiration; T&P, Temperature & Precipitation; SM, Soil Moisture; VPD, Vapor Pressure Difference; SR, Solar radiation; DEM, Digital Elevation Model; SPEI, Standardized Precipitation Evapotranspiration Index; LULC, Land Use and Land cover.

#### 2.2.2 Auxiliary data sets

In addition to the NPP data, several auxiliary datasets were used in this study, which include meteorological data, Land use and land cover (LULC) data, digital elevation model (DEM) data, and other relevant environmental factors. In this study, the meteorological data were obtained from the China Meteorological Data Sharing Service Center (https://data.cma.cn), covering the period from January 2001 to December 2020. This dataset includes daily mean temperature, maximum temperature, minimum temperature, and precipitation data from 11 meteorological stations within the study area (**Figure 1**), with detailed station information listed in **Table 1**. To generate continuous meteorological surfaces, Kriging interpolation was performed in ArcGIS 10.6. The interpolated raster data were then masked to the spatial extent of the MUSL, with pixel size and projection parameters consistent with those of the NPP dataset. Evapotranspiration data were derived from the MOD16A3 product, which provides annual global evapotranspiration estimates based on MODIS observations. Surface soil moisture data were sourced from the Global Surface Soil Moisture Dataset (1 km resolution) provided by the National Tibetan Plateau Data Center. Vapor pressure deficit (VPD) data were extracted from the TerraClimate dataset, which has a spatial resolution of 4.4km. Solar radiation data were obtained from the European Centre for Medium-Range Weather Forecasts (ECMWF), with a spatial resolution of 10 km. Additionally, the Standardized Precipitation Evapotranspiration Index (SPEI) on a 12-month timescale was retrieved from Xia et al. (2024).

LULC data were obtained from the National Land Use/Land Cover Monitoring Product (30 m resolution), developed by the Resource and Environmental Science and Data Center. The dataset, updated every five years, was used for the years 2000, 2010, and 2020. Land cover types were reclassified into six categories: cropland, forest, grassland, water bodies, construction land, and unused land. The DEM data were sourced from the Geospatial Data Cloud of China, with a scale of 1:50,000 and spatial resolution of 30 m. Slope and aspect information were derived from the DEM using ArcGIS tools, providing key topographic indicators for further analysis. An overview of all datasets and their corresponding metadata is provided in **Table 1**. To ensure consistency across all datasets, including LULC, DEM, and climatic variables, all raster data were subsequent modeling to match the spatial resolution (500 m) and coordinate system of the MOD17A3 NPP product. This preprocessing ensured spatial comparability in subsequent modeling and statistical analyses.

### 2.3 Methods

#### 2.3.1 Theil-Sen median estimator and Mann-Kendall test

The Theil–Sen median estimator and the Mann–Kendall (MK) test are widely used non-parametric methods for detecting trends in long-term time series. The Theil–Sen estimator calculated the median of all pairwise slopes in the series to estimate the trend slope, offering strong resistance to outliers and measurement errors (Wang et al., 2024b). The MK test is a rank-based statistical approach that detect the presence and significance of monotonic trends without requiring normally distributed data, and it is tolerant of missing values and tied ranks (Zhang et al., 2023). In this study, the Theil–Sen estimator was applied to quantify the magnitude and direction of temporal trends in annual NPP across the study area. The MK test was used to assess the statistical significance of these trends. Together, these methods provide a statistically sound framework for detecting and characterizing NPP dynamics under ecological restoration efforts.

#### 2.3.2 BFAST algorithm

The BFAST algorithm has been extensively applied to monitor vegetation change induced by forest disturbances and recovery (Fang et al., 2018). It is capable of detecting breakpoints in the annual NPP time series, and estimating the number, timing, and magnitude of these breakpoint. These breakpoints typically represent sudden shifts in the magnitude or direction of vegetation dynamics caused by external disturbances. BFAST decomposes an integrated time series into three components: long-term trend, seasonal variation, and residual fluctuation (Jiang et al., 2020b) (Equation 1). The model is expressed as:

(1) $$Y_{t}=T_{t}+S_{t}+\epsilon_{t},\;t=1,\cdot \cdot \cdot ,n$$

where, *Y_t_​* is the observed value at time *t*; *T_t_* is the trend component reflecting long-term structural changes; *S_t_* is the seasonal component representing intra-annual variability; and *ϵ_t_* is the residual capturing noise and unexplained variability.

**Figure 2:**
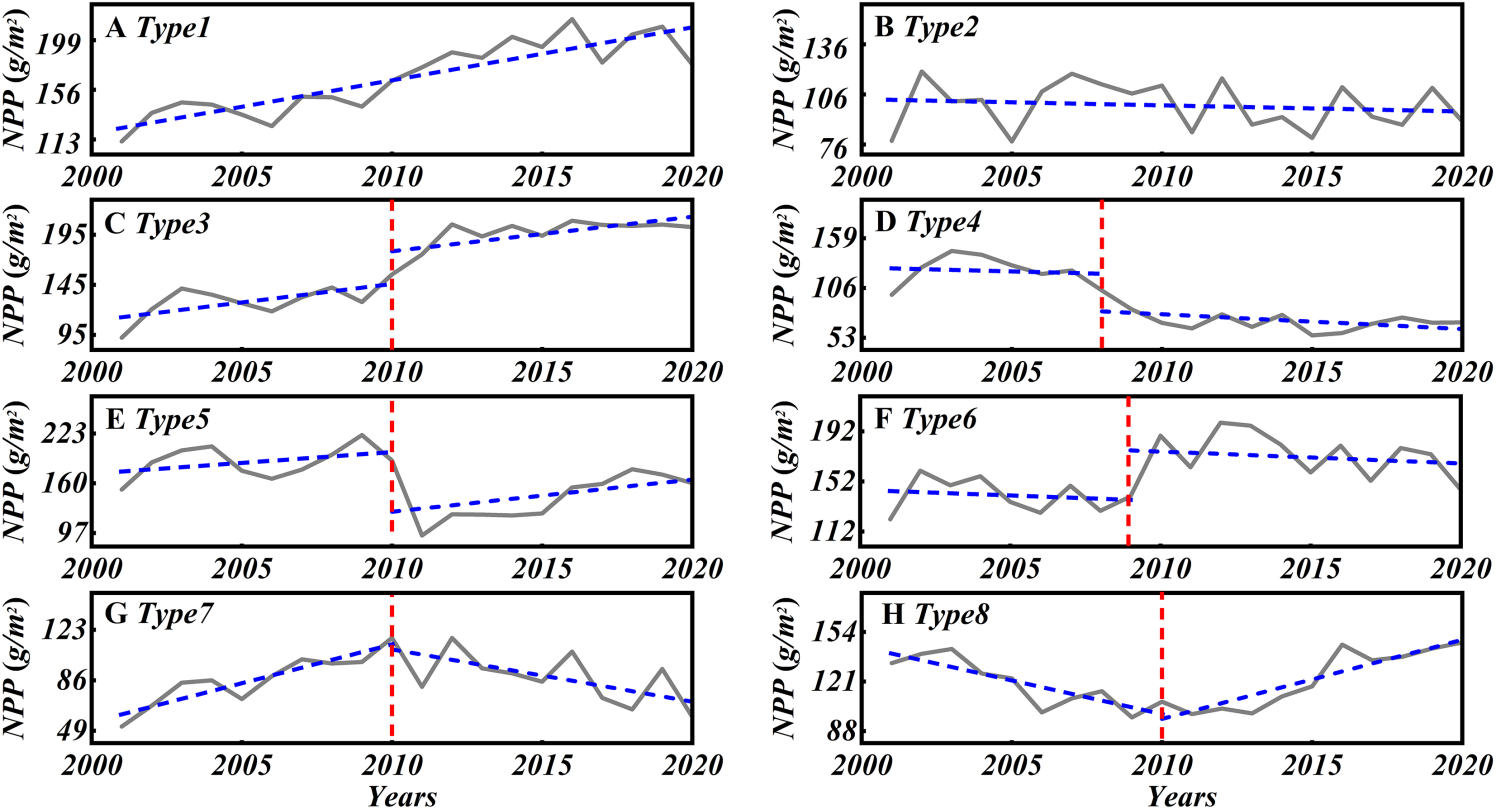
Schematic of eight NPP change patterns identified by the BFAST algorithm: **(A)** Type1–monotonic increase; **(B)** Type2–monotonic decrease; **(C)** Type3–increase with positive breakpoint; **(D)** Type4–decrease with negative breakpoint; **(E)** Type5–interrupted increase with negative breakpoint; **(F)** Type6–interrupted decrease with positive breakpoint; **(G)** Type7–increase followed by decrease; **(H)** Type8–decrease followed by increase.

In this study, BFAST01 was applied to annual NPP data to detect breakpoints in vegetation trends. Based on the implementation and shared code from Luo et al. (2022), the NPP time series for the MUSL from 2001 to 2020 were analyzed. The significance level for breakpoints detection was set to 0.05, and bandwidth values were incrementally used to enhance the reliability of the detected breakpoints. The shortest period for fitting the piecewise linear segments was set to 7 years, with other parameters set to their default values. During the analysis, to account for the significance of vegetation cover change trends, grids where both the pre- and post-segment trends were not significant were defined as having non-significant trends and classified separately. Following classification schemes adapted from Brakhasi et al. (2021) and Dou et al. (2024), the NPP trajectories were categorized into eight trend types, as illustrated in **Figure 2**.

#### 2.3.3 Structural equation modelling

**Figure 3:**
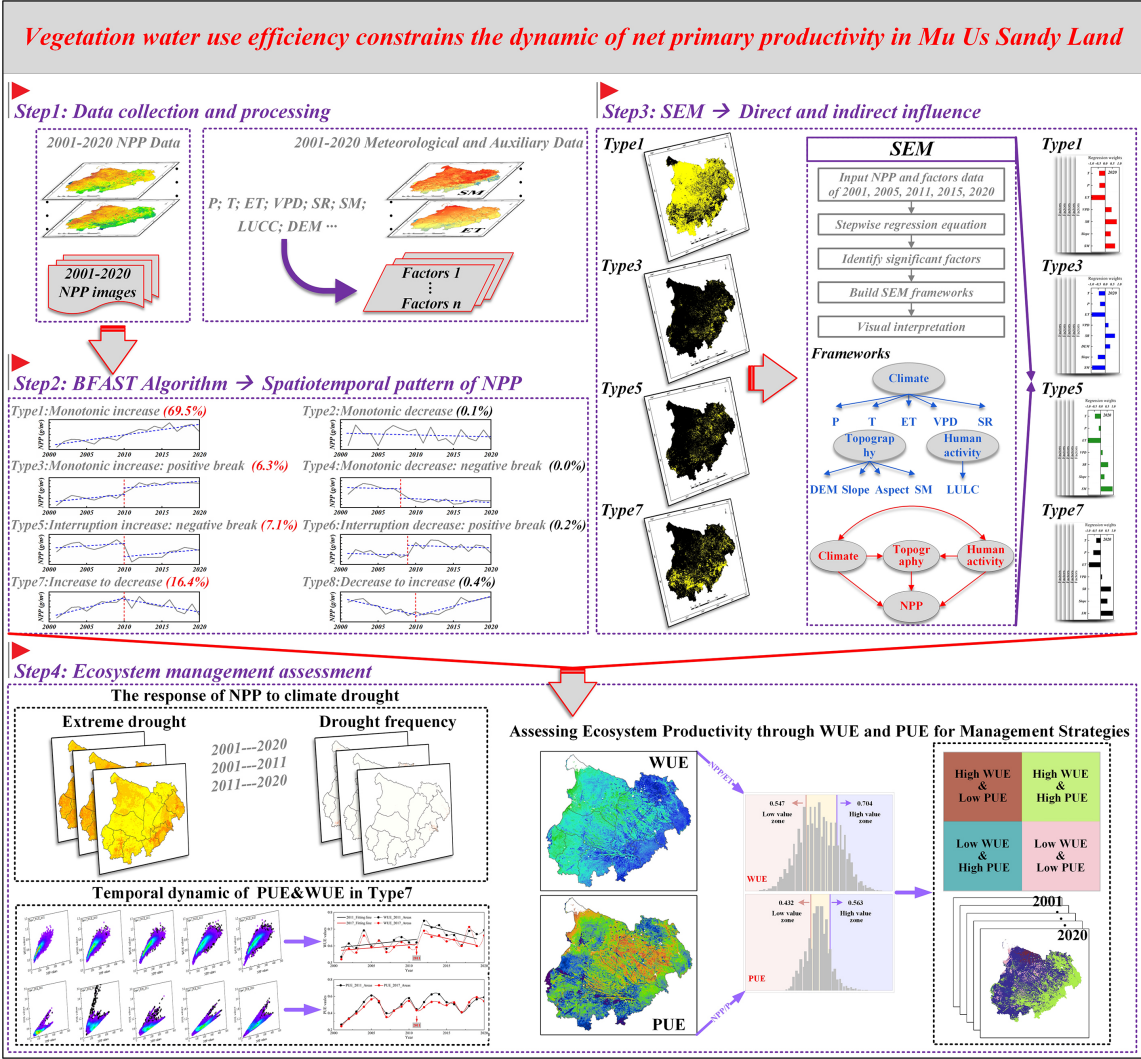
Schematic illustration of the overall workflow. Variable abbreviations are consistent with those in **Table 1**.

Structural equation modeling (SEM) integrates factor analysis and path analysis, allowing for simultaneous examination of complex relationships among multiple variables. It quantifies the direct, indirect, and total effects of independent variables on dependent variables (Zhang et al., 2024). In this study, AMOS software (ver.24.0; IBM SPSS, Inc.) was used to construct a model (**Figure 3**) to detect the influence of climatic factors (including P, T, ET, VPD, and SR), topographic factors (including DEM, slope, aspect, and SM), human activity (represented by LULC), and their synergistic effects on NPP variations (Feng et al., 2021), thereby revealing the main driving factors behind the spatiotemporal differences in NPP (detected by BFAST).

## 3 Results

### 3.1 Spatial characteristic analysis of environmental factors and LULC

**Figure 4:**
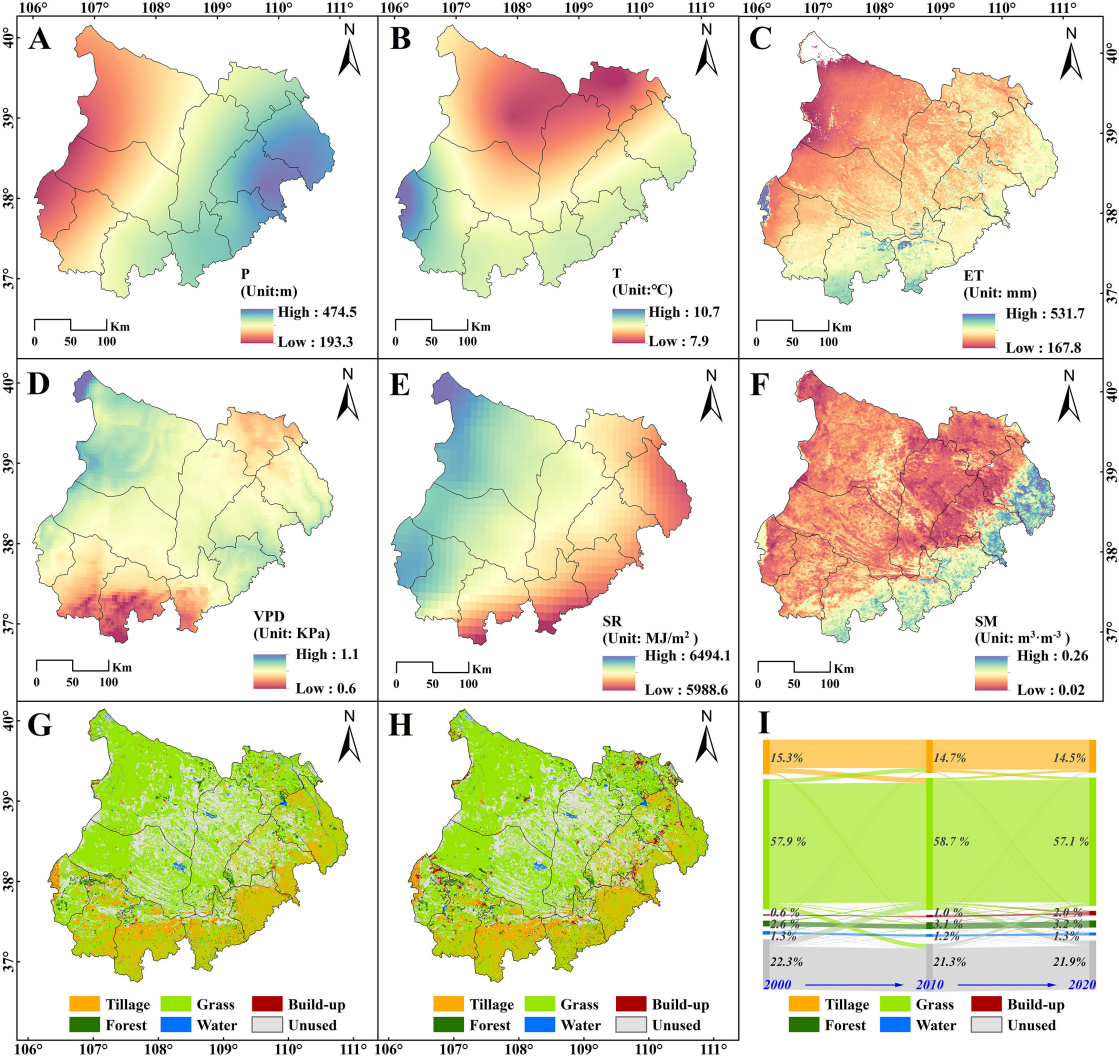
Spatial characteristic of environmental factors and LULC distribution. Specifically, it including **(A)** Precipitation-P; **(B)** Air Temperature-T; **(C)** Evapotranspiration-ET; **(D)** Vapor pressure Difference-VPD; **(E)** Solar radiation-SR; **(F)** Soil moisture at 0–8 cm depth-SM; **(G)** LULC distribution in 2000; **(H)** LULC distribution in 2020; and **(I)** temporal flow patterns of major LULC transitions.

**Figures 4A**-**F** illustrates the spatial distribution of key environmental and climatic variables in the MUSL from 2001 to 2020, including annual average precipitation (P), temperature (T), evapotranspiration (ET), vapor pressure deficit (VPD), solar radiation (SR), soil moisture (SM), and land use/land cover (LULC). Precipitation exhibited marked spatial heterogeneity (**Figure 4A**), increasing from northwest to southeast, ranging from 193 to 474 mm. The lowest precipitation (190–280 mm) was observed in the western MUSL, including Taole, Yinchuan, and Wuzhong, followed by moderate levels (280–380 mm) in central areas such as Dingbian, Yanchi, and Otog Banner. The highest precipitation (380–470 mm) was recorded in the eastern and southern subregions, including Yulin, Shenmu, Jingbian, Hengshan, and Ejin Horo Banner. In contrast, T followed a clear gradient, with colder conditions in the north and warmer conditions in the south regions (**Figure 4B**). The average temperature ranged from 7.9°C in the north to 10.7°C in the southwest, with an intra-regional variation of up to 2.8°C.

The annual average ET in the MUSL ranged from 160 mm to 400 mm, exhibiting a clear decreasing trend from southeast to northwest (**Figure 4C**). Most areas recorded ET values between 200 and 300 mm, while a small portion in the northwest showed values below 200 mm, accounting for approximately 4.5% of the total area. In contrast, southern regions exhibited ET values ranging from 300 to 350 mm, comprising about 10.3% of the area. Only a limited area—primarily cropland or forest—recorded ET values exceeding 350 mm, representing 1.3% of the region. **Figure 4D** shows the spatial distribution of VPD over the past two decades. The annual average VPD values ranged from 0.64 to 1.11 kPa, with lower values predominantly in the southern MUSL and higher values concentrated in the northwest and eastern regions. For SR, a southeast–northwest decreasing gradient was observed (**Figure 4E**), with annual values ranging from 5,988.6 MJ/m^2^ in the south to a maximum of 6,494.1 MJ/m^2^ in the northwest. The annual average SM at the 0–8 cm depth varied between 0.05 and 0.25 m^3^m^-3^ (**Figure 4F**). Most of the region exhibited SM values between 0.10 and 0.15 m^3^m^-3^. Lower values (<0.10 m^3^m^-3^) were mainly distributed in the central-northeast and western edge, while relatively higher values (0.15–0.20 m^3^m^-3^) were located in the eastern fringe, with some patches in the northeast exceeding 0.20 m^3^m^-3^.

The overall spatial pattern of LULC in the MUSL remained largely stable from 2000 to 2020 (**Figures 4G, H**). However, notable changes occurred within transitional zones between cultivated land, grassland, and unused land. Specifically, conversions from cultivated land to grassland were concentrated in Lingwu City, Yanchi County, and the eastern fringe, while grassland-to-cultivated land transitions occurred in Dingbian and Jingbian Counties. Grassland was converted to unused land in Wushen Banner, whereas the reverse occurred mainly in Otog and Otog Front Banners. The overlapping of these transitions led to significant land use shifts in southern areas such as Lingwu, Yanchi, and Dingbian (**Figure 4I**). Between 2000 and 2020, construction and forest land expanded, while grassland, cultivated land, unused land, and water bodies declined. Net area changes were: construction land (+1361 km²), grassland (–719 km²), cultivated land (–701 km²), forest land (+512 km²), unused land (–437 km²), and water bodies (–19 km²). Construction land grew the fastest—tripling in area—followed by forest land with a 21.6% increase. In contrast, grassland and water bodies showed minimal variation, both changing by less than 2%.

### 3.2 BFAST breakpoints detection for NPP

**Figure 5:**
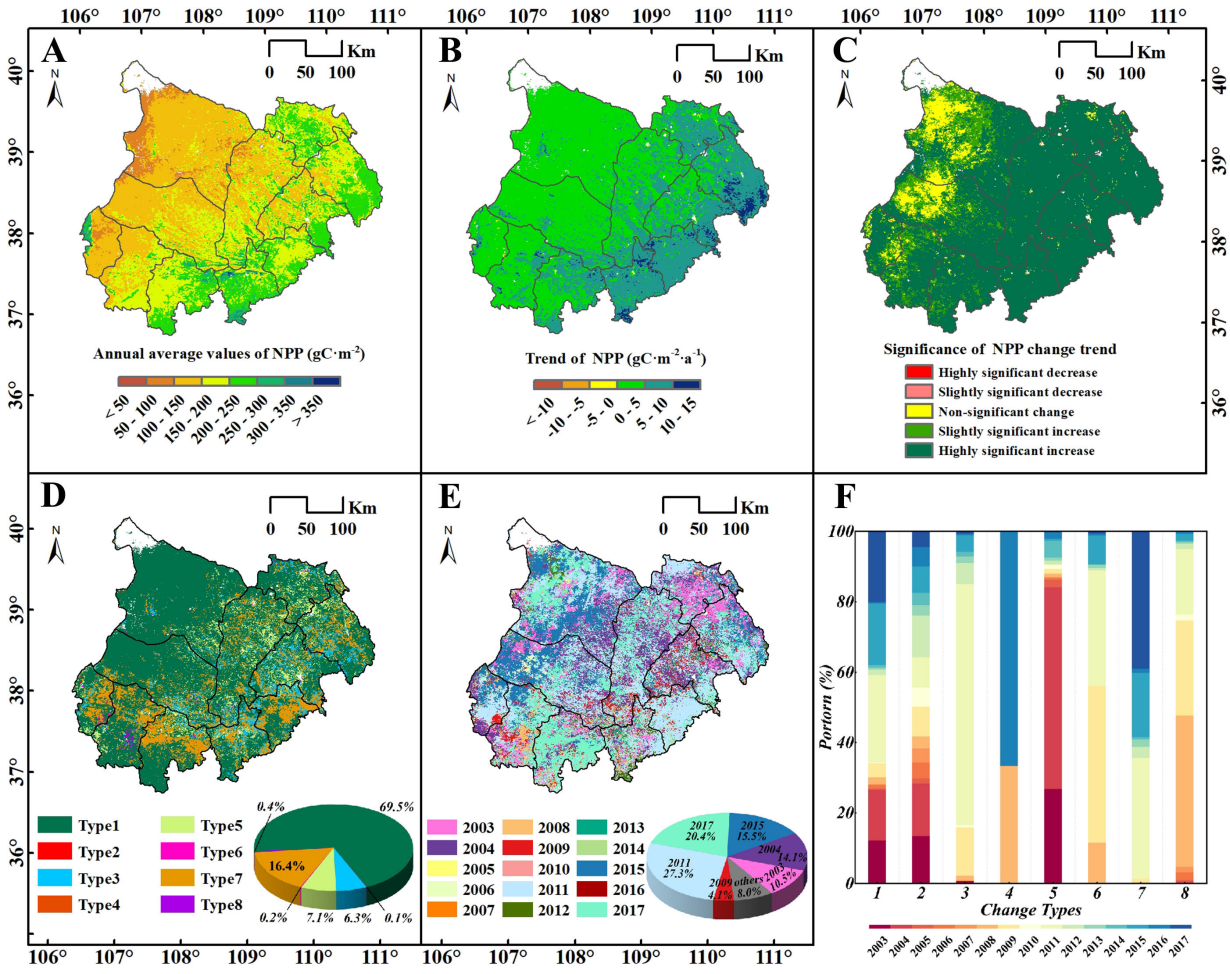
**(A)** Annual average, **(B)** trend, and **(C)** significance of NPP in the MUSL (2001–2020); **(D)** spatial distribution and **(E)** mutation years of eight NPP change types detected by BFAST; **(F)** proportion of mutation years across change types. Type 1–8 definitions are as in **Figures 2**.

The spatial distribution of annual average NPP values in the MUSL was shown in **Figure 5A**. The NPP high-value areas were predominantly located in the southeastern part of the MUSL, including Hengshan, Jingbian and Dingbian Counties, as well as the northeastern part of Ejin Horo Banner and Shenmu County. In these regions, most areas had NPP above 150 gC·m^-2^, with some areas exceeding 350 gC·m^-2^. In contrast, the low-value areas are primarily distributed in the western part, including Otog Banner, Otog Front Banner, and Lingwu City, where the NPP was mostly below 150 gC·m^-2^, with some areas less than 100 gC·m^-2^. The Theil–Sen median slope estimator, combined with the MK test was used to analyze pixel-level NPP trends in the MUSL from 2001 to 2020 (**Figures 5B, C**). Overall, NPP exhibited an increasing trend, with faster growth in the east and slower growth in the west. In the western and central regions, growth rates were mostly 0–5gC·m^-2^·a^-^¹, often with non-significant. In contrast, the eastern edge showed highly significant increases, with growth rates mostly between 5 and 10 gC·m^-2^·a^-^¹, and some areas exceeding 10 gC·m^-2^·a^-^¹.

The results of the BFAST01 detection in the MUSL from 2001 to 2020 differ somewhat from the results of the MK test (**Figures 5D, E**). Four change types (Type 1, 3, 5, and 7) account for a significant 99.3% of the total area (**Figure 5D**). Specifically, these types include: (1) Monotonic Increase (Type1), which accounts for 69.5% of the total area of ​​the total areas, widely distributed across the region, with the highest concentrated in the northwest. (2) Monotonic Increase with a positive break (Type3), accounting for 6.3%, mainly located in the southeast, particularly at the junction of counties and districts. (3) Interrupted Increase with a negative break (Type5), accounting for 7.1%, widely distributed in various areas outside the northwest. (4) Increase to Decrease (Type7), accounting for 16.4%, concentrated mainly in Dingbian, Hengshan, Lingwu, and Yanchi Counties, with a higher concentration in the southwest. In addition, small areas of Decrease to increase (Type8) and Interrupted decrease with a positive break (Type6) were observed in the southwestern part of the MUSL, accounting for 0.4% and 0.2%, respectively. Only a very small areas (only 0.1%) showed a Monotonic decrease (Type2), and there were virtually no areas with a Monotonic decrease with a negative break (Type4) in the MUSL.

From the perspective of temporal variability, breakpoints in the NPP time series across the MUSL occurred between 2003 and 2017, with the largest proportion appearing in 2011, accounting for 27.3% of all breakpoints and distributed across the entire region (**Figure 5E**). While the timing of breakpoints varied spatially, certain clustering patterns were evident. For instance, extensive areas in Otog Banner and Otog Front Banner experienced breakpoints in 2015, while most breakpoints in Dingbian County occurred in 2017. In contrast, breakpoints in the northeastern and central parts of the MUSL were mostly observed in 2003 and 2004, respectively. Furthermore, although the timing of breakpoints differed among NPP change types, the dominant breakpoint years of key types (Type 3, 5, and 7) coincided with the years of high breakpoint frequencies across the MUSL. Specifically: (1) For the Type7, breakpoints primarily occurred in 2011 and 2017. In 2011, breakpoints were concentrated in Lingwu and Hengshan County, while in 2017, they were primarily located in Dingbian, Shenmu, and Wushen Banner (**Figure 5F**). (2) For the Type5, breakpoints were concentrated in 2003 and 2004, together accounting for 84%, and were widely scattered across the region. (3) For the Type3, most breakpoints appeared in 2009 and 2011, with some scattered occurrences in Yuyang District in 2015.

### 3.3 Driving factor analysis for NPP

**Figure 6:**
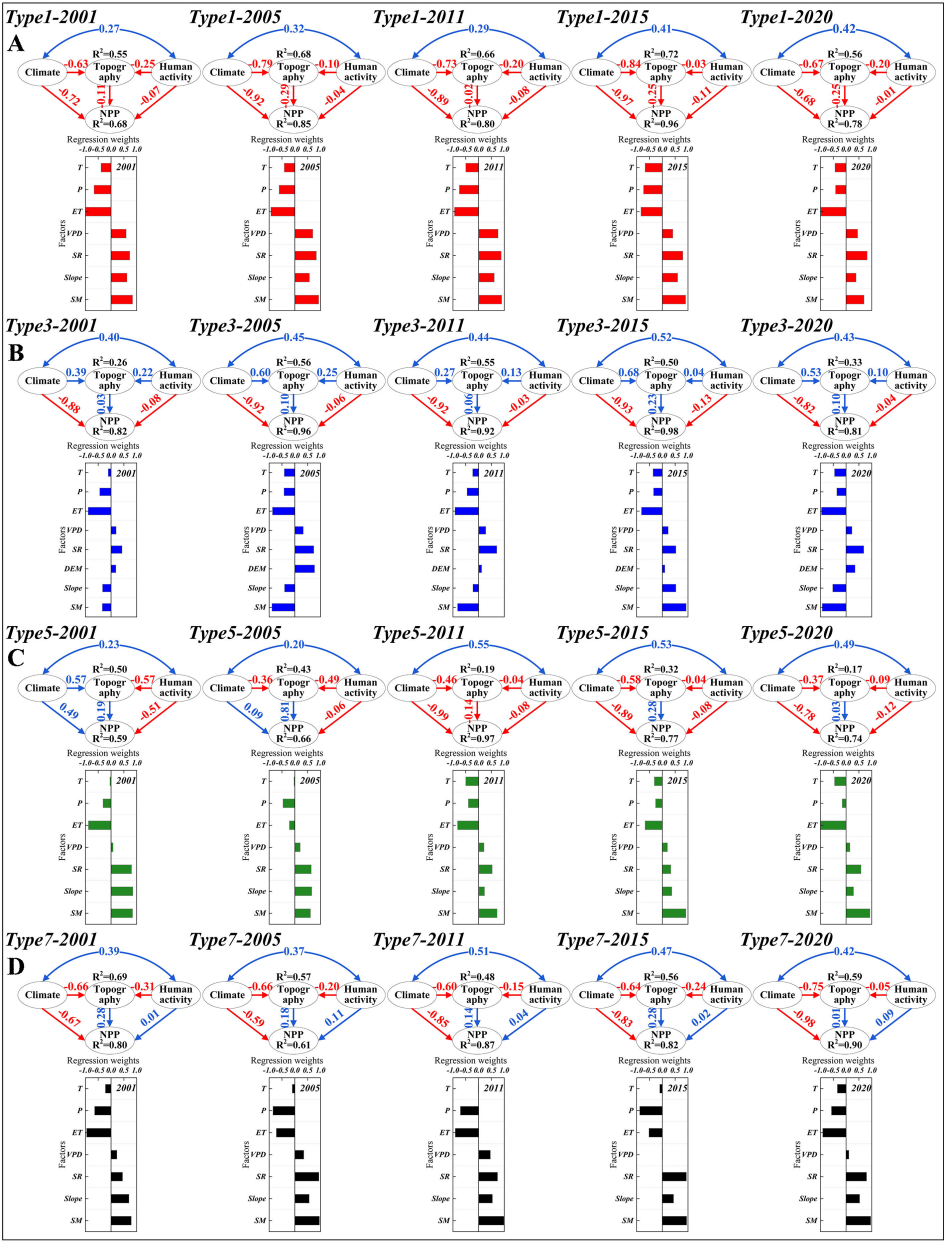
The direct effect, indirect effect, and regression weight of climate factors (P, T, ET, VPD, and SR), topography factor (DEM, slope, aspect, and SM), and human ecological restoration activity (LULC) on NPP in **(A)** Type1, **(B)** Type3, **(C)** Type5, and **(D)** Type7.

Based on the BFAST01-detected NPP change types and breakpoint years (**Figures 5D**-**F**), SEM was applied to analyze the responses of four major NPP types (Type1, Type3, Type5, and Type7) to meteorological factors (including P, T, ET, VPD, and SR), topographic factors (including DEM, slope, aspect, and SM), human ecological restoration activity (represented by LULC), and their synergistic effect. This approach aimed to identify environmental factors that promote or inhibit NPP increases across different regions of the MUSL (**Figure 6**). For Type1 breakpoints, BFAST01 detected an overall increasing trend without statistically significant shifts, with breakpoints mainly concentrated in 2001 (12%), 2005 (14%), 2010 (25%), 2015 (17%), and 2020 (20%) (**Figure 5F**). SEM results show that during these periods, T, P, and ET exerted strong positive effects on NPP (R^2^ = 0.68–0.96; standardized weights > 0.6), while VPD and SR had negative impacts (**Figure 6A**). This indicates that higher T, P, and ET facilitated NPP growth, whereas elevated VPD and SR suppressed it. In contrast, topographic factors and LULC also exerted negative effects, but with weaker path coefficients (< 0.3). Although the direct effect of ecological restoration activities remained weak (< 0.1), their synergistic influence with climatic drivers increased steadily from 0.27 in 2001 to 0.42 in 2020, suggesting a gradually strengthening indirect role of restoration efforts in supporting regional NPP under changing climate conditions.

For Type3 breakpoints, the majority occurred in 2011 (68%) (**Figure 5F**). As with Type1, P and T were the dominant climatic drivers of NPP (R^2^ = 0.81–0.98) from 2001 to 2020. Notably, the influence of P declined after 2011 (standardized weight decreased from 0.45 to 0.36), while the contribution of T increased (from 0.23 to 0.45). In comparison, topographic factors and ecological restoration activities had weaker effects, with path coefficients below 0.25—positive for the former and negative for the latter. The synergistic interaction between human activities and climate remained strong across the period, with coefficients consistently exceeding 0.4 (**Figure 6B**). For Type5, breakpoints mainly occurred in 2001 (27%) and 2005 (57%) (**Figure 5F**), during which NPP exhibited a sharp decline followed by a gradual recovery. SEM results indicate that this pattern was primarily driven by the direct effects of T, P, and ET, along with weak climate–restoration synergy (**Figure 6C**). In 2001 and 2005, although climatic factors showed a positive overall effect on NPP (R^2^ = 0.49 and 0.09, respectively), the regression weights of T, P, and ET were negative, suggesting these variables constrained NPP growth during this period. In subsequent years, the NPP development pattern of Type5 converged with that of Type1 and Type3.

Special attention was given to Type7, which exhibited a marked NPP decline over a substantial area (16.4%) in southern MUSL, with breakpoints concentrated in 2011 (34%) and 2017 (39%) (**Figure 5F**). SEM results revealed that from 2001 to 2011, climatic factors (P, T, ET) had a moderate direct-positive impact on NPP (R^2^ = 0.61–0.80; path coefficients = 0.59–0.67), and their synergy with ecological restoration efforts was relatively weak (0.37–0.39). In contrast, during 2011–2020, both the direct climatic influence (R^2^ = 0.82–0.90; coefficients = 0.83–0.98) and climate–restoration synergy (0.42–0.51) increased significantly (**Figure 6D**), with P and ET remaining the dominant contributing variables. Overall, the rapid increase in NPP over large areas of MUSL represented by Type1, Type3, and Type5 was primarily driven by the rapid and direct promoting effects of climatic factors such as P, T, and ET. In contrast, the rapid decline in NPP in the southern region of MUSL represented by Type7 after 2011 indicated that even with a high synergy between climatic factors and human ecological restoration activities, abrupt changes in climatic factors—such as an increase in P coupled with a decrease in ET, or vice versa—can still exert significantly impact on NPP. This highlighted the dominant role of climatic factors in influencing NPP changes.

### 3.4 NPP response to WUE, PUE, and SPEI

**Figure 7:**
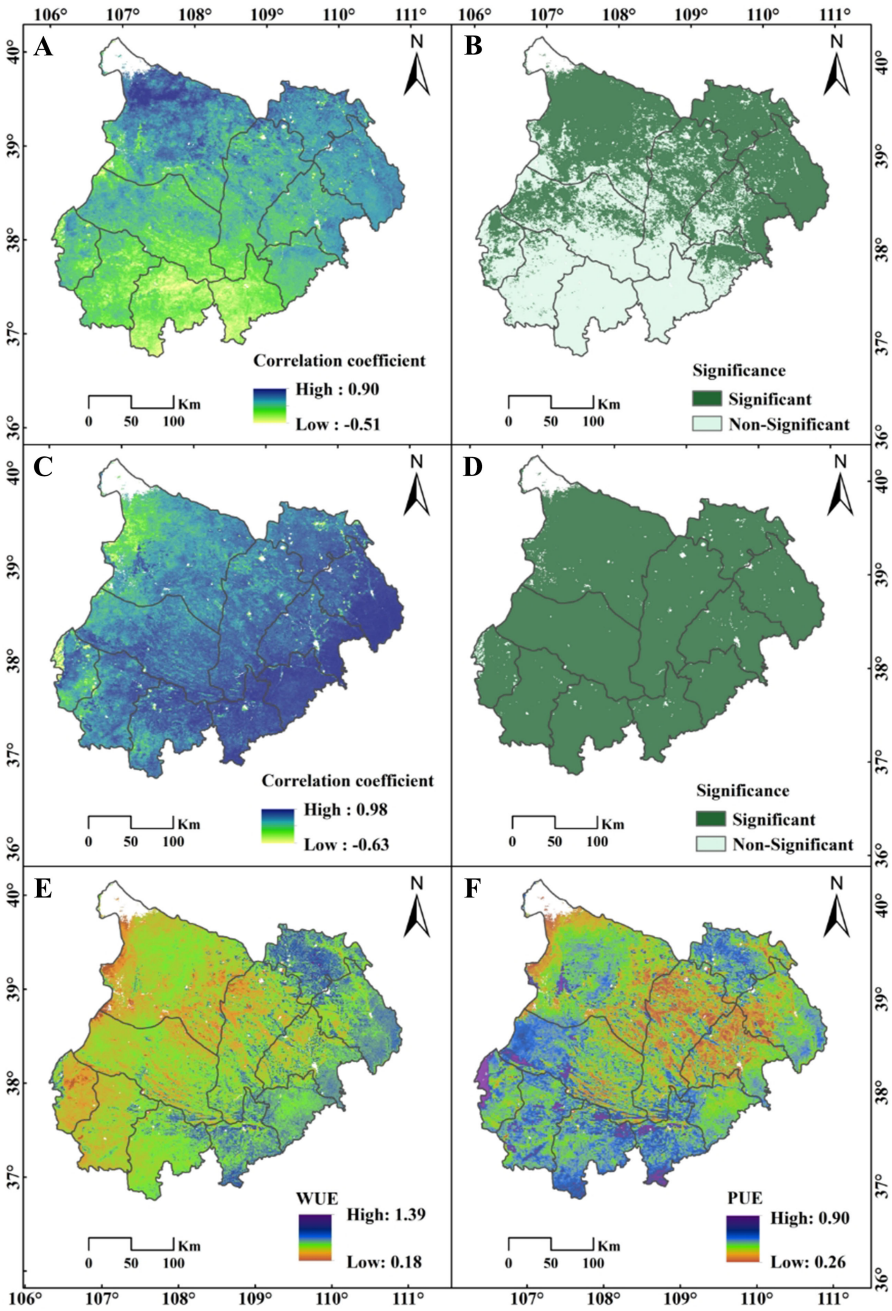
Spatial distribution of correlation coefficients between **(A)** NPP and P and **(C)** NPP and ET, with significant correlation (P<0.05) for **(B)** NPP and P and **(D)** NPP and ET. Annual average spatial distribution of **(E)** WUE and **(F)** PUE in the MUSL from 2001 to 2020.

**Figure 8:**
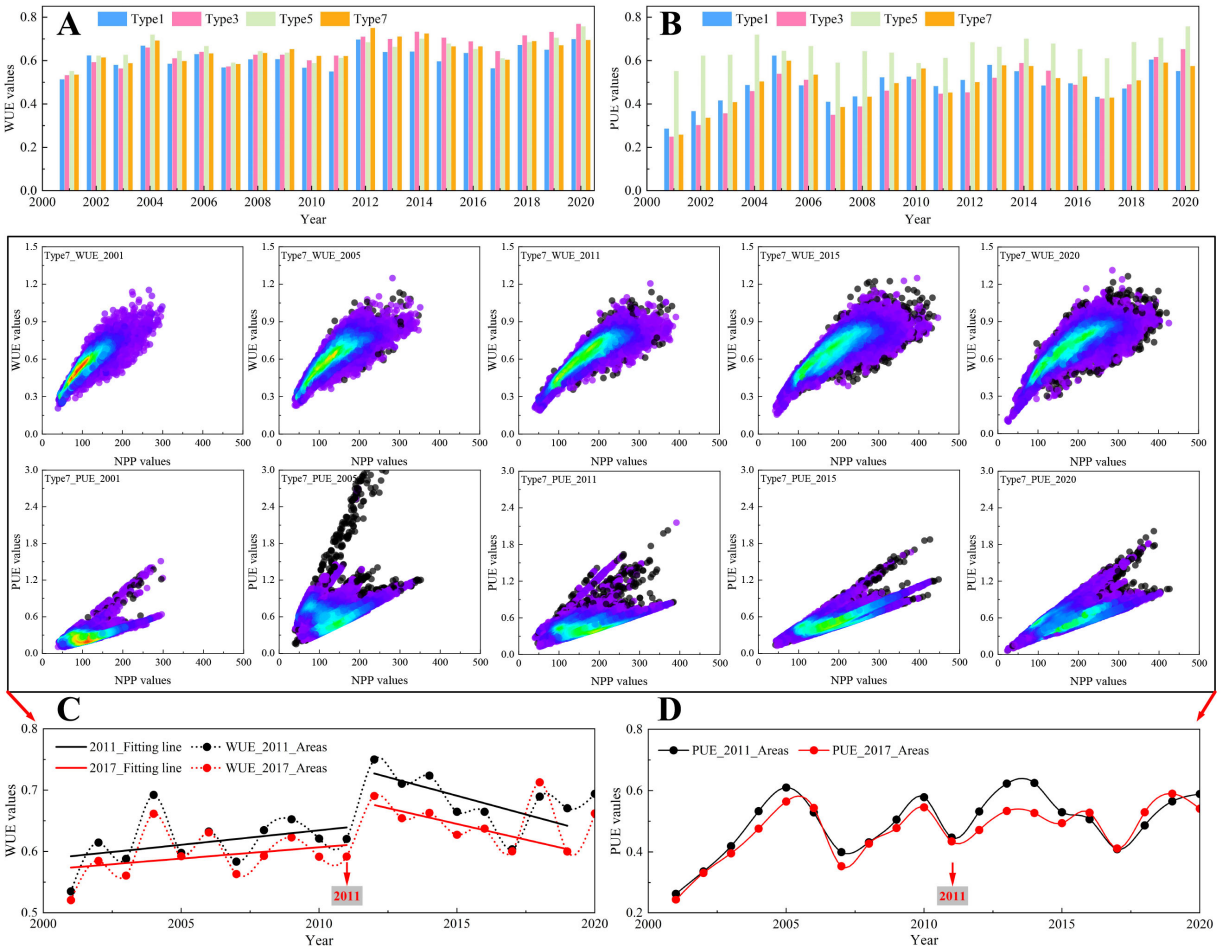
Variations of **(A)** WUE and **(B)** PUE in Type1, Type3, Type5, and Type7 regions. Scatter plots of WUE and PUE with NPP for Type7 in 2001, 2005, 2011, 2015, and 2020, with temporal dynamics of WUE and PUE extracted from Type7 areas in 2011 and 2017: **(C)** WUE trends and **(D)** PUE trends.

Building on the previous findings, the correlation analysis of NPP with P and ET suggested that precipitation may be the key factor driving the sharp NPP decline in the southern MUSL, as indicated by its weak and insignificant correlation (P < 0.05) (**Figures 7A–D**). To further assess the impacts of P, T, and ET on NPP trends, this section first employed the SPEI—incorporating both P and T—to evaluate extreme drought (**Supplementary Figures S1A–C**) and drought frequency (**Supplementary Figures S1D–F**) over 2001–2020 and two sub-periods (2001–2011 and 2011–2020). In addition, the relationships between NPP and both water-use efficiency (WUE = NPP/ET) and precipitation use efficiency (PUE = NPP/P) were explored across regions (**Figure 8**), providing insight into the effectiveness of current ecological management strategies in regulating NPP.

From 2001 to 2011, extreme drought was widespread across the MUSL, especially in the southern (S3, S4, S8) and eastern regions (S1, S2, S5, S11), with events concentrated in 2005 and 2008 and SPEI values dropping below –2 (**Supplementary Figure S1C**). Drought frequency during this period exceeded 10 events in the eastern areas, including S11, S2, S1, S5, and S4 (**Supplementary Figure S1D**). Between 2011 and 2020, extreme drought intensity declined, mostly affecting southern and eastern regions with SPEI values around –1.5 (**Supplementary Figure S1E**). Southern droughts were concentrated in 2010, 2012, 2015, and 2017, while eastern events occurred in 2015, 2017, and 2019. Overall, drought frequency decreased to fewer than five events across most regions, indicating notable mitigation (**Supplementary Figure S1F**).

**Figure 9:**
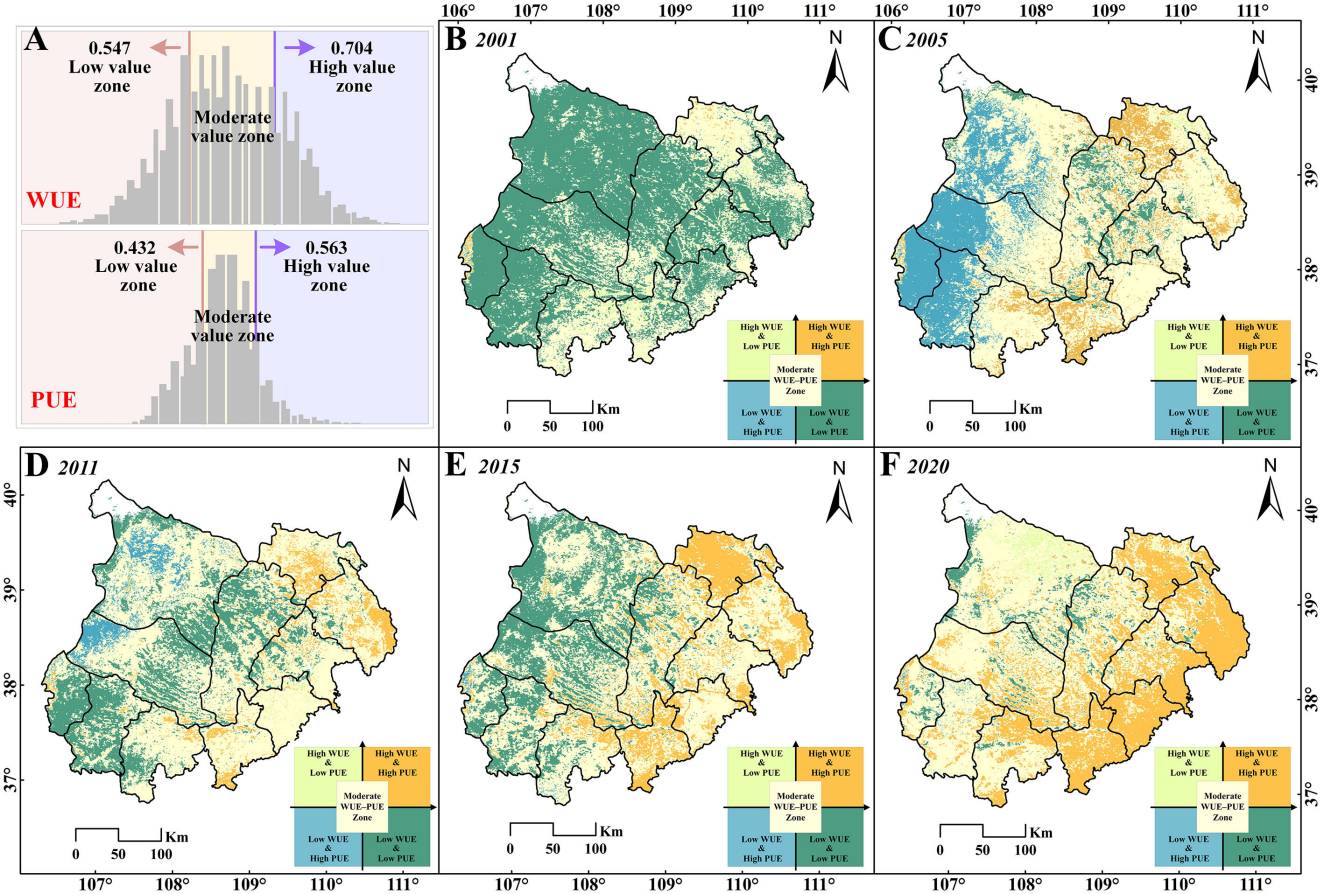
WUE and PUE quartile-based classifications in the MUSL from 2001 to 2020. **(A)** Frequency histograms of classified zones. **(B–F)** Spatial distributions of combined WUE–PUE categories in 2001, 2005, 2011, 2015, and 2020.

**Figures 8A, B** illustrates the temporal dynamics of WUE and PUE for Types 1, 3, 5, and 7, along with changes in Type 7 regions that experienced breakpoints in 2011 and 2017 (**Figures 8C, D**). From 2001 to 2011, Type 7 showed the highest WUE among all types (**Figure 8A**), steadily increasing and peaking at 0.75 in 2012. Thereafter, WUE declined sharply, reaching a decade-low of 0.6 by 2017 (**Figure 8C**). In contrast, PUE exhibited clear five-year cyclical fluctuations, ranging between 0.3 and 0.6 (**Figure 8D**). Meanwhile, **Figures 7E, F** and **Figure 9** illustrates the spatial distribution of WUE and PUE from 2001 to 2020, categorized by quartiles to identify high- and low-value regions. This classification provides insights into how ecological management strategies have influenced NPP growth from the perspective of water resource efficiency. In 2001, both low WUE–low PUE and moderate WUE–moderate PUE areas were widely distributed across the MUSL (**Figure 9B**), indicating generally low utilization efficiency of precipitation and available water resources (including irrigation, runoff, and groundwater), thereby reflecting deficiencies in water management practices. By 2005, a significant portion of low WUE–low PUE areas shifted to low WUE–high PUE and moderate WUE–moderate PUE areas, while high WUE–high PUE zones began to emerge in the southern and northern MUSL (**Figure 9C**). This transition suggests more effective use of precipitation in the central and western subregions; however, overall water resource efficiency remained suboptimal, continuing to constrain NPP growth. In contrast, 2011 marked a renewed expansion of low WUE–low PUE areas, especially in the northern and central MUSL (**Figure 9D**), with the trend intensifying through 2015 (**Figure 9E**). This pattern suggests that in the absence of substantial management improvements, vegetation water use efficiency may have reached a threshold, contributing to NPP degradation (**Figure 8A**). By 2020, however, a notable shift occurred as low WUE–low PUE areas transitioned into moderate WUE–moderate PUE zones, and high WUE–high PUE regions expanded markedly, particularly in the eastern MUSL (**Figure 9F**). These changes reflect substantial gains in both WUE and PUE, likely driven by the adoption of more effective ecological and agricultural management practices. Overall, while eastern MUSL has achieved significant progress in water resource utilization, large areas remain characterized by recurrent transitions between low and moderate efficiency states.

## 4 Discussion

### 4.1 The critical role of breakpoint analysis in revealing NPP development patterns

In arid regions, NPP is a key indicator for assessing ecosystem productivity and the outcomes of ecological restoration efforts (Kang et al., 2019). This study used the BFAST01 algorithm to identify four primary NPP evolution patterns and breakpoint types (**Figures 2**, **5**), providing a more detailed perspective on ecosystem dynamics and structural transformation. The results show that from 2001 to 2020, NPP development patterns dominated by Type 1, Type 3, and Type 5 (accounting for 69.5%, 6.3%, and 7.1% of the total area of the MUSL, respectively) exhibited significant growth trends (**Figures 5D-F**), consistent with findings from several studies of Yan et al. (2015); Wang et al. (2023a) and Dou et al. (2024). However, despite the overall increasing trend in most of the MUSL, the southern region (accounting for 16.4% of the total area) exhibited a specific degradation pattern (Type 7, Increase to Decrease) in 2011 and 2017 (**Figure 5D**), which has rarely been mentioned in previous studies. The primary cause of this pattern is that ecosystem responses to environmental factors are typically non-linear and multi-phased (Chen et al., 2025b). Traditional trend analysis methods, such as the Theil–Sen median estimator, MK test, and EEMD, generally focus on identifying the overall development trend of NPP across the entire region (Fang et al., 2018; Dou et al., 2024), but fail to effectively capture local changes and breakpoint features. This may result in the overlooking of potential declining trends in NPP at different stages of development (Liu et al., 2025). In particular, in the MUSL, where climate change is complex and the effectiveness of ecological restoration fluctuates, the spatial clustering of these NPP breakpoints reflects the negative impact of climate change and restoration practices on NPP increases (Qi et al., 2023). Ignoring breakpoints in trend analysis may obscure the actual dynamic changes in NPP in the region, where significant declines in NPP are present in certain areas, leading to an inability to accurately capture the non-linear characteristics of NPP (e.g., increase to decrease, monotonic decrease, and decrease with negative breakpoint), which in turn causes misinterpretations of ecosystem dynamics at different stages of development (Mardian et al., 2021; Liu et al., 2025). This finding challenge overly optimistic interpretations based solely on average trends and reveals potential degradation risks. Therefore, integrating trend and breakpoint analyses into multi-temporal assessments is crucial for accurately capturing NPP trajectories and effectively evaluating restoration outcomes.

### 4.2 The synergistic effect of climate factors and human ecological restoration activity on NPP

The impact of climatic factors and LULC on NPP has been widely studied (Xu et al., 2020; Mu et al., 2024). However, previous studies have often focused on the separate effects of human activities or climate change on ecosystems, with significant debate over their relative contributions (Chen et al., 2024b; Zhu et al., 2024). For example, Gang et al. (2018) in their study on the Loess Plateau showed that the effects of human activities and climate change accounted for 78.45% and 21.55%, respectively. In contrast, Gao et al. (2022) found that human activities and climatic factors had similar impacts on NPP, with human activities having a greater influence (56.44%). In contrast, the SEM results of this study revealed significant spatial variations in the synergistic effects of climatic factors and LULC on NPP (**Figure 6**), which have increased over time, from 0.27 in 2001 to 0.42 in 2020. In particular, in the southern part of the MUSL, represented by Type 7 (Increase to Decrease), the synergistic effect (0.42–0.51) between climatic factors and human activities was higher than in regions with NPP growth patterns (Type 1, Type 3, and Type 5). However, this strong synergy may be insufficient to offset the negative impacts of abrupt climate changes or weak vegetation responses in the southern MUSL (Wang et al., 2017; Yan et al., 2019). Specifically, after 2011, the path coefficients for climatic factors in the southern region (ranging from 0.83 to 0.98) were much higher than for LULC and topographic factors, with P having a significant regression weight (**Figure 6D**). Correlation analysis between NPP and precipitation revealed that P had a low, non-significant correlation in the southern MUSL (**Figures 7A, B**). We speculate that this may be related to the duration of ecological restoration projects, vegetation density, and species characteristics, as previous studies by Zhang et al. (2022) and Lu et al. (2024) have shown that mature vegetation in restoration projects is often more sensitive to drought stress. Furthermore, the SPEI results indicate that since 2011, the southern and eastern parts of the MUSL have continued to experience relatively severe extreme drought events compared to other regions (Wang et al., 2023b). Here, analysis of WUE and PUE further suggests that exceeding the threshold of water use efficiency may be another key factor contributing to the decline in NPP in the southern region. Therefore, this study confirms that the trend in NPP variation is primarily driven by climate change, including both its direct effects on NPP and its synergistic interaction with human activities.

Although the direct effects of LULC and topographic factors on NPP growth are limited, this low direct influence does not negate their importance, particularly in the positive role of ecological restoration projects in increasing regional greening (Xu et al., 2024; Zhao et al., 2024). In this context, the impact of LULC is more evident in the synergistic interaction between climatic drivers and ecological restoration efforts. This is consistent with Zhang and Wu (2020), who argued that vegetation restoration in the MUSL is primarily driven by improvements within land types, rather than conversions between them. Our LULC change analysis supports this view: prior to 2000, ecological protection policies such as returning farmland to forest and grassland, along with afforestation and aerial seeding, achieved significant success (Jiang et al., 2017; Han et al., 2020). From 2000 to 2010, cropland decreased by 502 km^2^, while grassland and forest areas increased by 733 km^2^ and 415 km^2^, respectively, and unused land decreased by 966 km^2^ (**Figures 4G-I**). However, from 2010 to 2020, water scarcity in the semi-arid areas began to limit the sustainability of restoration efforts (Zhang and Wu, 2020; Zheng et al., 2020). Due to water shortage, grassland established through aerial seeding decreased by 1452 km², while irrigated forests saw slight gains (+96 km^2^). Although cropland continued to decrease, the reduction (198 km^2^) was smaller than in the previous decade. At the same time, urbanization led to rapid expansion of construction land. The LULC flow pattern from 2000 to 2020 (**Figure 4I**) further illustrates significant internal transformations in the southern MUSL, especially the shift towards grassland (Wu et al., 2024). This indicates that although these changes have limited direct effects on NPP, they may increase ecosystem vulnerability and contribute to NPP decline (Lv et al., 2021). Therefore, from the perspective of long-term sustainability, the intrinsic role of LULC in shaping ecosystem dynamics warrants further investigation (Zhao et al., 2024). In conclusion, by evaluating the impacts of different environmental factors (both individually and interactively), and extracting the direct, indirect, and synergistic effects of climatic factors and LULC on vegetation NPP growth, this study provides a better understanding of the driving mechanisms behind the monotonic growth trend of NPP and identifies effective measures to address climate and human activity changes.

### 4.3 Priority management regions for ecological restoration and countermeasures

**Figure 10:**
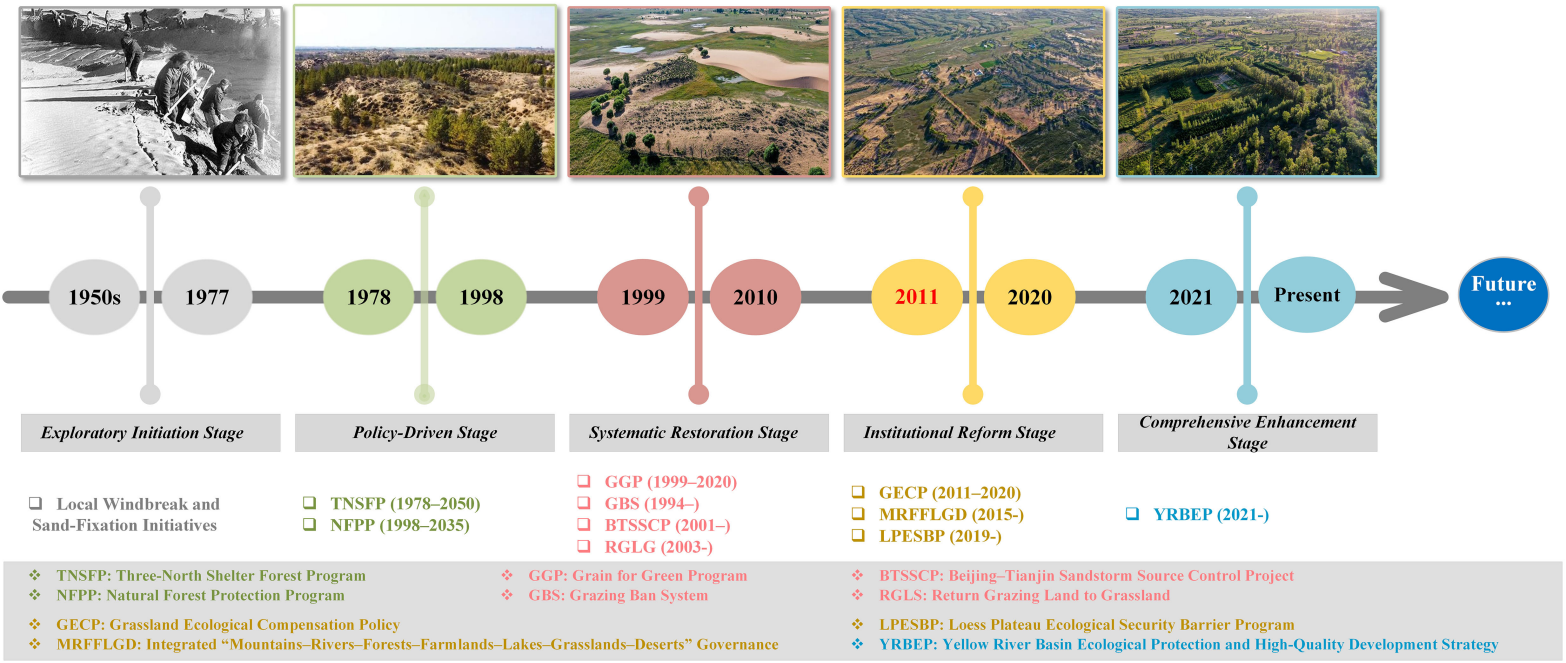
Timeline of ecological restoration stages and policy evolution in the MUSL.

The year 2020 marked a critical evaluation point for several major ecological restoration initiatives in the MUSL, including the “Three-North Shelter Forest Project” (1978–2050), “Natural Forest Protection Program” (1998–2020), “Grain for Green Project” (1999–2020), and “Grazing Ban Policy” (1996–2020) (**Figure 10**) (Jiang et al., 2020a; Wang and Li, 2022). Assessing the effectiveness of these projects through the lens of water use efficiency is essential for evaluating restoration outcomes, summarizing practical experience, and guiding future ecological protection strategies (Hu et al., 2023; Liu et al., 2023). Despite notable restoration achievements, significant challenges persist, particularly in the southern MUSL, where NPP has declined continuously since 2011, affecting 16.4% of the area. As discussed previously, two key factors contribute to this decline: (i) the high synergy between climatic factors and ecological restoration efforts is insufficient to offset the adverse impacts of extreme drought and low vegetation responsiveness to precipitation; and (ii) water use efficiency (WUE) may have reached its functional threshold in these regions. Here, there is a clear indication that the water use efficiency across most of the MUSL is unstable (**Figure 9**). Specifically, there are only the eastern region demonstrates a consistent transition from low to moderate and ultimately high WUE–PUE levels between 2001 and 2020, supported by favorable hydrothermal conditions and effective use of water resources (including irrigation, runoff, reservoirs, and groundwater). In contrast, most other regions—including the north, west, center, and south—oscillated repeatedly between low and moderate WUE–PUE levels over time (2001→2005→2011→2015→2020). As mentioned by Xu et al. (2025) and Chen et al. (2024a), this instability is primarily due to arid conditions and dominance of drought-tolerant shrubs and semi-shrubs, with sparse presence of grasslands, forests, or crops. Although both WUE and PUE are low, increasing precipitation over the years has buffered NPP decline, with NPP trends largely driven by the cyclic behavior of PUE (He et al., 2024). In contrast, in the southern MUSL, although hydrothermal conditions are relatively favorable, WUE and PUE remain at moderate levels, indicating suboptimal and unstable water use. As such, intensified drought or land use changes may trigger renewed or accelerated NPP declines (Gang et al., 2019). Meanwhile, this is all supported by the weak NPP–P correlation (**Figures 7A, B**), the spatial distribution of post-2011 extreme drought events (**Supplementary Figures S1E, F**), LULC transition patterns (**Figure 4**), and the threshold behavior of WUE (**Figures 8A, C**). These findings underscore that current restoration efforts and water management strategies may be insufficient to support future ecological development needs, necessitating targeted adjustments.

Given these findings, several targeted management strategies are proposed to support sustainable development in the next phase. First, the southern MUSL should be designated as a priority zone for ecological restoration, with a focus on introducing more drought- and cold-tolerant vegetation (Chen et al., 2024a). This is especially critical in view of the increasing frequency of extreme droughts projected for the region, which coincides with areas showing weak and insignificant NPP–P correlations and the highest concentration of drought events (**Supplementary Figure S1**). In these zones, the conventional aerial grass seeding approach should be phased out in favor of natural vegetation succession involving drought-resilient shrubs and semi-shrubs. Prior research by Chen et al. (2023) indicates that grasslands exhibit lower water retention capacity than shrubs or bare land, and dense grass seeding may lead to excessive depletion of soil moisture and groundwater, ultimately causing vegetation mortality and reduced biomass productivity. Furthermore, as suggested by the observed threshold behavior of WUE in the southern MUSL (**Figures 8A, C**), optimizing the composition and planting density of trees, shrubs, and grasses is necessary (Xu et al., 2020; Wang et al., 2025). In particular, balancing age structure—especially the proportion of mature versus juvenile vegetation—may enhance water use partitioning, increase overall water use efficiency, and reduce vulnerability of vegetation productivity to climate extremes (Gang et al., 2019; Chen et al., 2025a).

### 4.4 Limitation and uncertainties

It should be noted that there are some limitations and uncertainties regarding the attribution of vegetation NPP increase and its sustainability in this study. First, although this study considered above-ground influencing factors on NPP changes, including climatic factors, topographic factors, human ecological restoration activities, and their synergistic effect, the consideration of human activity-related factors (such as population density, agricultural activities, and socio-economic development levels) was not comprehensive enough. Future research should focus on the comprehensive relationship between these factors and vegetation changes. Second, this study has not yet clarified whether the vegetation water use efficiency reaching its threshold is caused by increased vegetation density or by an improper planting ratio (the ratio of shrub, grass, and tree species or the management of old-age and young-age trees). As Shan et al. (2025) reported, productivity stability decreases with forest age, making it crucial to explore this issue further for a deeper understanding of NPP variations. Additionally, underground factors, such as root-zone soil moisture and groundwater, were not included in this study. The dynamic changes in soil moisture and groundwater are also crucial indicators reflecting the dynamic changes in NPP (Huang and Shao, 2019; Jiang et al., 2019). In the future, we aim to address these limitations and uncertainties and assist decision-makers in the scientific and rational management of regional ecosystems.

## 5 Conclusions

This study combined the BFAST01 algorithm, structural equation modeling, and WUE threshold analysis to detect abrupt shifts in NPP and disentangle the relative roles of climatic variability versus human-induced restoration. The main conclusions are as follows:

1. Ecological restoration success and challenges: Although ecological restoration in the MUSL has been highly successful, with 82.9% of the area showing an increasing NPP trend, challenges remain—particularly in the southern region with more favorable hydrothermal conditions, where NPP has continuously declined since 2011, impacting 16.4% of the area.
2. Primary divers of NPP decline: While the synergistic effects of climatic factors and ecological restoration efforts have significantly promoted NPP growth in most regions, this synergy proves insufficient in the south region of MUSL. In this region, the limited vegetation response to precipitation and the threshold effect of WUE are the primary drivers of the observed decline. Spatiotemporal analyses of WUE and PUE reveal that large portions of the MUSL remain in a suboptimal state, oscillating between low and moderate efficiency zones. These regions, especially under current planting and management strategies, remain vulnerable to future drought or intensified LULC changes—risks that could replicate the declining NPP trend observed in the south and potentially make it irreversible elsewhere (excluding the eastern region).
3. Recommendations for future restoration: To mitigate these risks and enhance ecosystem resilience, future restoration should prioritize: (i) abandoning the traditional aerial grass seed sowing mode; (ii) reducing the grass seed density; (iii) introducing drought-tolerant native shrubs in the south; and (iv) optimizing the composition and age structure of tree–shrub–grass systems. Overall, this study provides a scientific foundation for improving ecological restoration strategies and supports sustainable development planning in arid and semi-arid ecosystems such as the MUSL.

## Data Availability

The original contributions presented in the study are included in the article/**Supplementary Material**. Further inquiries can be directed to the corresponding authors.

## Supplementary material

Supplementary Figure 1 Spatial distribution of **(A, C, E)** extreme drought and **(B, D, F)** drought frequency in MUSL for the periods 2001-2020, 2001-2011, and 2011-2020.
