# Supplementary figures and images for: Vegetation water use efficiency constrains the dynamic of net primary productivity in Mu Us Sandy Land
Source: Front Plant Sci. 2026 Feb 4;17:1724283. doi: 10.3389/fpls.2026.1724283 (PMC12913464; doi:10.3389/fpls.2026.1724283)

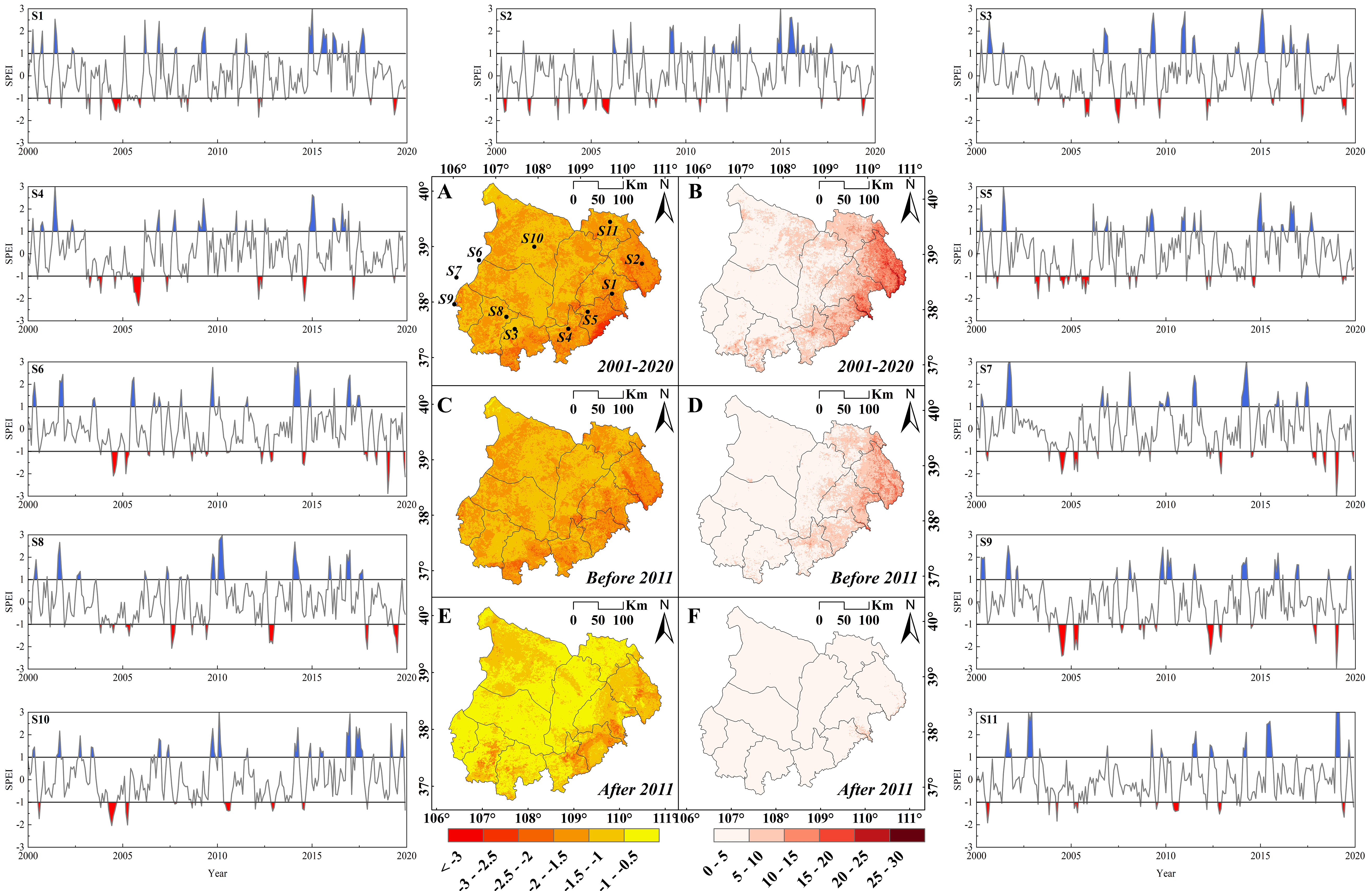

Supplement: Supplementary Figure 1 — Spatial distribution of (A, C, E) extreme drought and (B, D, F) drought frequency in MUSL for the periods 2001-2020, 2001-2011, and 2011-2020. [file Image1.jpeg]
